# Giant cell arteritis: a cause of temporomandibular joint claudication

**DOI:** 10.25122/jml-2022-0076

**Published:** 2022-10

**Authors:** Eric Chun-Pu Chu, Rick Chiang

**Affiliations:** 1 New York Chiropractic and Physiotherapy Centre, Hong Kong SAR, China; 2 School of Health and Rehabilitation Sciences, The University of Queensland, Brisbane, Australia

**Keywords:** chiropractic therapy, giant cell arteritis, jaw claudication, ARIA – Autonomic retinal image analysis, CT – Computed tomography, GCA– Giant cell arteritis, TMJ– Temporomandibular joint

## Abstract

The purpose of this case report was to benefit the clinical recognition and conservative management of giant cell arteritis (GCA) in temporal arteries associated with jaw claudication. Giant cell arteritis is a systemic inflammatory vasculitis that affects medium-to-large-sized arteries. Primarily affecting arteries in heads, especially in temples, chronic GCA can result in secondary headaches and even polymyalgia rheumatica. This is a case report of a 68-year-old female with a 10-year history of GCA. The patient presented jaw claudication, headache, and joint stiffness over 6 months. The left palpable superficial temporal artery was thickened and tendered. A full-spine radiograph revealed uneven shoulders, imbalanced jaws, and moderate lumbar scoliosis. After nine months with conservative management, the patient was completely recovered from the symptoms with significantly improved radiographic parameters. Patients with GCA can present with jaw claudication. Physiotherapy and chiropractic collaborations are options for patients with GCA who suffer from the chronic adverse effect of medicines. Clinicians should be aware of the common clinical findings associated with GCA when rehabilitation treatment is planned.

## INTRODUCTION

Giant cell arteritis (GCA), frequently referred to as temporal arteritis, is a systemic inflammatory vasculitis that affects medium- to large-sized arteries, mostly in temples [1]. GCA occurs at a rate of 29.6 cases per 100,000 individuals, in 70–79 years old [2]. The major arteries involved in GCA are the medium-sized muscular arteries, including the cranial and extracranial branches of the carotid artery [1]. As highly prevalent in elders, GCA accounts for a significant portion of headaches in older adults [1]. Furthermore, GCA is closely associated with polymyalgia rheumatica (PMR), a chronic inflammatory rheumatic disorder with unknown causes [1], given that a substantial number of patients exhibit GCA and PMR together [3].

The GCA classification criteria by the American College of Rheumatology [4] include 1) onset age of 50 years or greater, 2) new-onset or localized headache, 3) tenderness or decreased pulsation in at least one of the temporal arteries, 4) erythrocyte sedimentation rate of 50 mm/h or greater, and 5) biopsy of an abnormal artery with mononuclear cell infiltrate or granulomatous inflammation [4]. A diagnosis of GCA requires meeting at least three of the five criteria, and we present a patient who fits in the criteria by meeting four characteristics, except for a biopsy examination. Despite the paucity of biopsy tests due to the patient's refusal, the present case is diagnosed as GCA given the notable symptoms, also supported by previous reports of frequent failure of GCA biopsy due to the focal and segmental nature of the infiltrates resulting in normal histological examination in approximately 15% of GCA cases [5]. Our case represents the first report describing non-pharmacological management of chronic GCA patients whose symptoms failed to improve by conventional treatments.

## CASE REPORT

A 68-year-old Asian woman with well-controlled hypertension presented with six months of jaw claudication, headache, and temporomandibular joint (TMJ) stiffness. The aching sensation began in the left jaw while chewing and gradually worsened over the last three months. The persistent TMJ claudication resulted in abstaining from solid foods, followed by morning stiffness in the shoulders, neck, and low back. She has a 10-year medical history of chronic headaches diagnosed with GCA and suffered from osteoporosis due to chronic steroid use. Her headache was widespread but the most intense in her right temporal region. Exposure to cold made the headache worse. Although migraine prophylaxis and analgesics were ineffective, steroid therapy improved symptoms within 48 hours. She attempted to wean herself off medication but frequently relapsed over the years.

### Investigation

A dentist and a neurologist examined the patient and ruled out dental, cranial, and neurological disorders. Despite the no evidence of ischaemic lesions in her brain CT, the patient's blood profile revealed an elevated erythrocyte sedimentation rate and C reactive protein levels. She declined a biopsy procedure due to previous failures. Although re-treatment alleviated some of the symptoms, she experienced recurrences of additional complaints such as TMJ, cervical, lumbar, and shoulder pain. To address her musculoskeletal pain, the patient was referred to chiropractic and physiotherapy.

**Figure 1:**
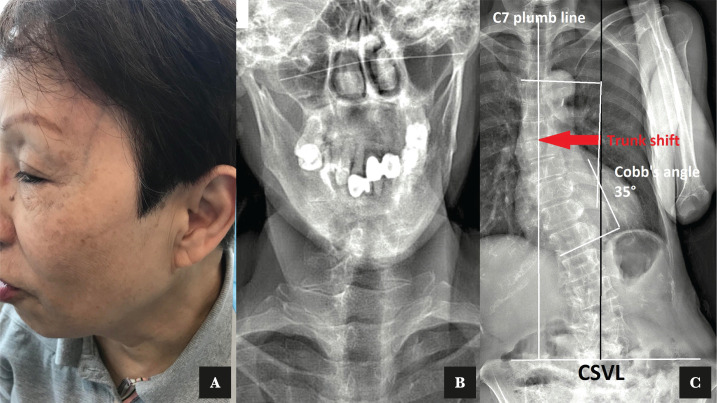
A – Photography of a 68-years old female with giant cell arteritis with the thickened and palpable left superficial temporal artery was seen. B – Uneven coronoid process and mandibular condyle. C – Standing full spine EOS® coronal images showed dextro-convexity of the lumbar curve with associated musculoskeletal complaints.

The patient presented with a thickened temporal artery upon examination (Figure 1 A). Her cardiovascular examination revealed no abnormalities. Neck and joint pain were rated as 6/10 on a numeric pain scale. Orthopedic exams of the TMJ were negative. Manual muscle testing revealed bilateral upper trapezius hypertonicity, cervical extensor hypertonicity, and right temporalis muscle hypertonicity with a noticeable grating sensation. At the cervical extension, active neck ranges of motion were limited by ten degrees, and intervertebral joint dysfunction was identified at the C2-3, C4-5, C5-6, T1-2, and T6-7 levels. EOS^®^ imaging of the pre-treatment posture revealed a right deviation of the jaw, uneven shoulders, lumbar scoliosis, and multiple levels of vertebral wedging with osteophytosis (Figure 1 B and C).

### Diagnosis

In the absence of neurological deficits and the biomechanical functions of joints that appeared to be interfered with by the severe joint pains, the patient was diagnosed with myofascial pain syndrome secondary to GCA.

### Treatment

We performed chiropractic and physiotherapy consisting of spinal manipulation of the restricted cervical segments, thermal ultrasound therapy and intermittent motorized traction of the cervicothoracic spine. The treatment regime is widely applied in chiropractic practice to relieve neck stiffness, restore normal joint motion, release intervertebral spaces and decompress neural impaction. Treatment sessions were arranged three times weekly for two months.

The initial phase of treatment focused on posture correction and pain reduction. To ensure vascular health throughout the treatment process, stroke risk was screened biweekly using autonomic retinal image analysis (ARIA). Spinal manipulative therapy, heat ultrasound therapy, and soft tissue treatment were used to relieve tight muscles and restore joint function in the upper cervical, TMJ, and paraspinal muscles.

### Outcome

**Figure 2:**
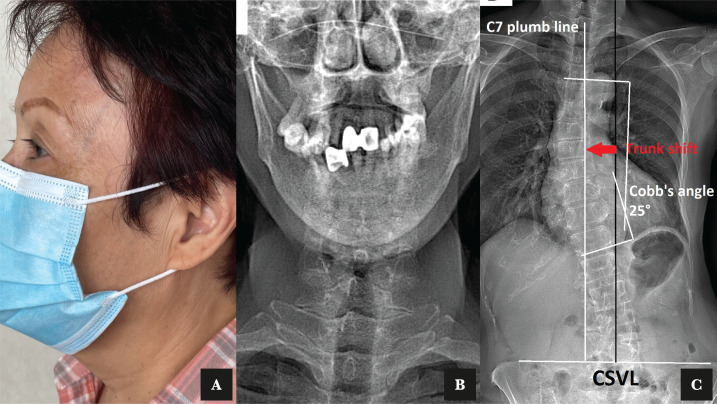
A – Repeat photography after treatment showed a normalization of the superficial temporal artery. B – Repeat radiograph after chiropractic care showed a normalization of the jaw. C – Repeat radiographs after treatments showed a reduction of lumbar scoliosis.

Treatment sessions were scheduled three times a week for two weeks. The patient reported that her headache and jaw symptoms improved gradually at the second visit and mostly resolved by the end of two weeks. The prominence of the superficial temporal artery was significantly reduced after two weeks of treatment (Figure 2 A). The patient continued to enter the corrective stage weekly for the next three months, with an emphasis on improving head posture and spinal misalignment. Repeat EOS^®^ radiographs (Figure 2 B and C) at the ninth-month follow-up revealed improvement in her shoulder balance, TMJ alignment, and lumbar scoliosis. After fifteen months, she reported being symptom-free with no difficulty chewing. She did not report any adverse events.

## DISCUSSION

Prompt treatments for ischemia are essential in preventing blindness and other ischemic complications, so clinicians should be well aware of the associated signs and symptoms and refer patients appropriately. Frequently, vascular complications result from a delay in diagnosis and treatment initiation [6]. In literature, the most effective management strategy for ischemia entails the use of glucocorticoids [6]. Unfortunately, GCA is a chronic systemic disease with a variable course and duration, so most patients taper and discontinue steroids after a few years [6]. Moreover, certain medications requiring long-term administration may develop into chronic conditions. Likely, patients with long-lasting glucocorticoid treatment frequently suffer from serious side effects [6]. There is no known GCA case study on the management of chronic patients who did not respond to medication in the PubMed databases. Although it is uncommon for GCA to increase the risk of ischemic stroke [7], clinicians should be aware of the common clinical findings associated with GCA when rehabilitation treatment is planned.

After our thorough examinations of associated risk, conservative rehabilitation was administered to patients with GCA who suffered from the adverse effect of steroid treatment. To assess the vascular risk in our patient, autonomic retinal image analysis (ARIA) [8] was adopted with daily blood pressure monitoring in collaboration with the neurologist. As a non-invasive photograph-based assessment for the retina, the ARIA confidently analyzed the retinal blood vessels and provided vital information about vascular health. This is further supported by previous studies demonstrating over 95% sensitivity of ARIA in estimating stroke risk and cardiovascular health [8]. We also performed daily monitoring of bilateral blood pressure in search of a predominating unilateral vascular stenosis that provided updated information on cardiovascular conditions throughout the recovery period [9].

The pathogenesis of GCA has not been clearly understood. Numerous hypotheses have been proposed, including autoimmune, genetic, environmental, and ischemia-induced destruction of the muscular layers of the artery's tunica media [10]. Some previous studies suggested that GCA's clinical characteristics are influenced by the autonomic nervous system [10]. There was a case of temporal arteritis with a sympathetic component in the orofacial region [11]. The headache and orofacial pain were well-managed by stellate ganglion blocks that decreased noxious peripheral stimulation and central pain transmission [11]. Another study suggested that parasympathetic dysfunction caused by ciliary ganglion ischemia could occasionally result in the mydriatic pupil as a manifestation of GCA [12]. Chiropractic manipulation and rehabilitation are known to treat neck tongue syndrome [13], regulate the autonomic nervous system and its projections to the central nervous system [14]. Similarly, spinal manipulation has biomechanical effects on symptom reduction through muscle relaxation and nerve release [15]. Thus, conservative rehabilitation may be considered an additional treatment option for GCA patients who do not respond well to traditional medications and suffer from musculoskeletal pain.

As this is a case report, it would be necessary to confirm the present observation in a large patient cohort. Given the limited facility at our chiropractic clinic, we used EOS^®^ radiographs for both TMJ and full spine. EOS^®^ full spine radiographs likely exhibit differences from a standard dental radiograph in the assessment of TMJ that must be considered in the comprehensive radiographic evaluation. Regardless of those limitations, this case report may add fundamental knowledge of GCA related to applying modified treatments to meet the needs of patients with coexisting risks.

## CONCLUSION

Patients with GCA can present with jaw claudication. The management of patients with GCA is considered complicated and requires a multifaceted treatment strategy involving a multidisciplinary collaboration of the symptoms in relation to its physiology, anatomy, and functions. Clinicians should be aware of the common clinical findings associated with GCA, not only for diagnosis but also for critical and long-term rehabilitation.

## Data Availability

Further data are available from the corresponding author upon reasonable request.
